# One-week inpatient cognitive behavioral therapy for insomnia: a retrospective study

**DOI:** 10.3389/fpsyt.2024.1440026

**Published:** 2024-08-27

**Authors:** Qing Cai, Mengya Li, Guifeng Li, Xin Xu, Cheng Li, Qifang Li

**Affiliations:** Department of Anesthesiology and Perioperative Medicine, Shanghai Key Laboratory of Anesthesiology and Brain Functional Modulation, Clinical Research Center for Anesthesiology and Perioperative Medicine, Translational Research Institute of Brain and Brain-Like Intelligence, Shanghai Fourth People’s Hospital, School of Medicine, Tongji University, Shanghai, China

**Keywords:** insomnia, sleep disorder, CBT-I, inpatient, retrospective study

## Abstract

**Objective:**

To examine the effectiveness of one-week inpatient cognitive behavioral therapy for insomnia (CBT-I) in patients without severe mental disorders in the real-world setting to answer the research question “Can inpatient CBT-I be abbreviated?”.

**Methods:**

In this retrospective, single-group, pretest–posttest study, the clinical outcome data of 94 patients who underwent one-week inpatient CBT-I were collected. Self-report scale scores and hypnotic medication use were obtained at baseline and at the 3-month follow-up after therapy.

**Results:**

CBT-I significantly improved insomnia severity (*Z* = −7.65, *P* < 0.001, Cohen’s *d =* 1.34), anxiety (*Z* = −6.23, *P* < 0.001, Cohen’s *d =* 1.02), depression (*Z* = −6.42, *P* < 0.001, Cohen’s *d =* 1.06), daytime sleepiness (*Z* = −2.40, *P* = 0.016, Cohen’s *d =* 0.35), and fatigue severity (*Z* = −5.54, *P* < 0.001, Cohen’s *d =* 0.88) and reduced hypnotic medication use (*χ^2^* = 33.62, *P* < 0.001). At the follow-up assessment, 58 patients (67.4%) had clinically meaningful changes in insomnia, and 51 patients (59.3%) met the criteria for insomnia remission.

**Conclusion:**

The results of this preliminary study imply that one-week inpatient CBT-I may be an effective intervention for the treatment of insomnia in patients without severe mental disorders.

## 1 Introduction

Insomnia is characterized by difficulty falling asleep, difficulty maintaining sleep, and/or waking up earlier than intended, and it is the most common sleep disorder affecting 9%–15% of the general population (1, 2). Insomnia is associated with daytime symptoms, such as impaired alert cognition, mood disturbance, sleepiness, and fatigue, which markedly affect quality of life (3).

Cognitive behavioral therapy for insomnia (CBT-I) is considered a first-line treatment for insomnia in adults and has been recommended as the first choice in the European region (4, 5). Traditional CBT-I is delivered by a specialized clinician in the outpatient setting through individual face-to-face sessions over a period of 6–8 weeks. Nevertheless, the shortage of trained clinicians and the high cost of treatment restrict the availability of individual face-to-face CBT-I (6, 7). In China, service obstacles and financial difficulties constitute significant barriers to accessing mental health services (8). There were 44,943 licensed psychiatrists and psychiatric registrars (3.2/100,000 population) by the end of 2020 (9). Only 4.5% of patients with mental disorders have sought help from mental health professionals (10).

To overcome these barriers, researchers have created various delivery formats for CBT-I, such as group, telephone, Internet, self-help, and inpatient formats, to make CBT-I more accessible to patients (11–13). A systematic review showed that the effect of synchronously delivered CBT-I (individual and group therapy on site) is better than that of other delivery formats (13). In addition, “abbreviated” CBT-I can save time and reduce costs by shortening the treatment duration or employing non-specialists to perform the therapy, but may reduce the treatment effect (14–16). The stepped-care strategy categorizes CBT-I into varying levels of treatment intensity based on the severity and complexity of insomnia to efficiently treat as many patients as possible with limited resources (17, 18).

Inpatient CBT-I is often used to treat insomnia in hospitalized patients with severe mental disorders (19). Due to the fact that patients can be closely coached by specialized clinicians, inpatient CBT-I has good efficacy and acceptability. Crönlein et al. (2014) demonstrated that 14-day inpatient CBT-I may benefit patients with general insomnia who are unable to access or who do not respond to outpatient CBT-I (11). Inpatient CBT-I may become an important option in the management of stepped-care. Nevertheless, the high cost of weeks of hospitalization hinders the widespread use of inpatient CBT-I. In addition, there is no research on the effectiveness of short-term (less than 2 weeks) inpatient CBT-I for patients with insomnia without severe mental disorders.

Therefore, we developed a one-week inpatient CBT-I program with a particular focus on low cost and offered it to patients at our department. This program allowed the CBT-I providers to treat more patients in a shorter period of time. The objective of this study was to examine the effectiveness of this one-week inpatient CBT-I program for patients with insomnia without severe mental disorders in the real-world setting to answer the research question “Can inpatient CBT-I be abbreviated?”

## 2 Materials and methods

### 2.1 Study design and participants

This was a retrospective, single group, pretest-posttest study. We reviewed the medical records of patients who visited the Department of Anesthesiology and Sleep Medicine of Shanghai Fourth People’s Hospital for sleep disturbance between May 1, 2023 and January 31, 2024. The Institutional Review Board of Shanghai Fourth People’s Hospital, Shanghai, China approved the study on April 15, 2024 (File No. 2024068–001; Chairperson, Qisheng Zhang). Because this study was retrospective, we were unable to obtain informed consent. Informed consent was waived by the institutional review board.

The inclusion criteria were as follows: age 18–75 years; fulfillment of the diagnostic criteria for chronic insomnia disorder per the third edition of the International Classification of Sleep Disorders (20); and received the one-week inpatient CBT-I program. The exclusion criteria were as follows: incomplete baseline data; another sleep disorder (e.g., sleep apnea syndrome, restless legs syndrome, circadian rhythm disorder); severe mental disorder (e.g., schizophrenia, bipolar disorder, major depressive disorder); shift work; and participation in other interventions for insomnia. Patients were not excluded if they were taking hypnotics, antidepressants, or antipsychotics prior to treatment.

Patients were self-referred or referred to the CBT-I program by their psychiatrist, neurologist, or sleep specialist. They were assessed at baseline and followed up at 3 months after the one-week inpatient treatment.

### 2.2 Interventions

This one-week inpatient CBT-I program was adapted and developed from the 14-day inpatient protocol outlined by Crönlein et al. (2014) (11). We shortened the hospitalization duration, eliminated polysomnography, and did not offer individual sessions. The CBT-I program consisted of group sessions, behavioral therapy supervised and guided by a sleep specialist, and a fixed bedtime and wake-up time schedule.

A group of 6–10 patients was admitted and discharged on the same day. The patients were assigned to two-, three- or eight-person bedrooms on the ward, depending on sex. The bedroom temperature was maintained between 20°C and 23°C while the patients slept. The patients were provided with soft earplugs to mitigate the effects of noise.

The group CBT-I sessions started on the first day of the treatment, which included the five classic modules of CBT-I (sleep restriction, stimulus control, relaxation training, cognitive therapy and sleep hygiene). The sessions were provided once a day for 6 days, with each session lasting for 60–90 minutes. The co-author (ML), who is a trained CBT-I therapist with 3 years of experience, facilitated the sessions as the main therapist. The content of each session is presented in **Table 1**. Sleep restriction started on the second day of treatment. To facilitate ward management, the time in bed for all patients was set at 6 hours per night during hospitalization. The patients were permitted to go to bed between 23:00 and 0:00, and they were not permitted to take daytime naps. Relaxation training started on the third day of treatment. A trainee CBT-I therapist led the patients in abdominal breathing exercises and progressive muscle relaxation twice per day (morning and evening). A sleep specialist provided daily supervision and guidance to the patients.

**Table 1:** Content of CBT-I sessions.

Session	Content
1	Psychoeducation and overview Psychoeducation on circadian rhythms, sleep structure, and causes of insomnia. Introduction to the inpatient CBT-I program and treatment duration. Setting personal treatment goals. Introduction to filling out sleep diaries and the calculation of sleep efficiency.
2	Sleep restriction and stimulation control Introduction to the effects and principles of sleep restriction and stimulation control. Setting time in bed. Full explanation of how to adjust time in bed based on sleep efficiency.
3	Relaxation training Introduction to the purpose and principles of relaxation training. Demonstration of abdominal breathing exercise and progressive muscle relaxation by CBT-I therapist. Setting the duration and frequency of relaxation training.
4	Cognitive therapy Explanation of common misconceptions about insomnia and sleep (e.g. insomnia may lead to sudden death, I need to sleep for 8 h per day, and I must be asleep by 22:00). Establishing correct thoughts and beliefs.
5	Sleep hygiene Introduction to the environmental settings and lifestyle changes that can improve sleep (e.g. reducing caffeine and alcohol intake and avoiding exercise before bedtime).
6	Review and post-discharge reminders Review the previous content. Reminder to follow behavioral therapy as required for at least 6 weeks after discharge, to add stimulus control when want to end sleep restriction, and to maintain sleep hygiene practices even after sleep has returned to normal.

CBT-I, cognitive behavioral therapy for insomnia.

Patients taking hypnotics were recommended to discontinue these medications gradually. The sleep specialist educated the patients about the symptoms and duration of the withdrawal reaction, and the pace of dose reduction was 10%–25% of the initial dose every 2 weeks (21). The patients were permitted to slow the discontinuation if they were experiencing significant withdrawal symptoms. Patients taking antidepressants or antipsychotics were asked to continue taking these medications.

After discharge, the patients were permitted to reset their bedtime and wake-up time according to their lifestyle. They were asked to continue behavioral therapy for at least 6 weeks and adjust their time in bed weekly to achieve a target sleep efficiency of 85%–90%. The minimum time in bed was set at 5 hours per night.

### 2.3 Measures

#### 2.3.1 Demographic characteristics

Patients’ demographic characteristics were collected from their medical records, including sex, age, marital status, level of education, working status, and medications.

#### 2.3.2 Insomnia Severity Index (ISI)

The ISI is a seven-item self-report scale that assesses the severity of insomnia over the past 2 weeks, which has good reliability and validity (22). Each item is rated from 0 (none) to 4 (high severity) on a five-point Likert scale, with the total score ranging from 0 to 28. In this study, Cronbach’s alpha for ISI was 0.88 at baseline and 0.91 at follow-up.

A decrease of 8 points in the ISI score was used to determine whether a patient was a treatment responder (23). Insomnia remission was defined as a post-treatment ISI score of <8 points.

#### 2.3.3 Hospital Anxiety and Depression Scale (HADS)

The HADS is a self-report scale for measuring anxiety and depression (24). It consists of two seven-item subscales, including HADS-Anxiety (HADS-A) and HADS-Depression (HADS-D). The total score for each subscale ranges from 0 to 21, with a higher score indicating more severe anxiety/depression. Cronbach’s alpha was 0.87 at baseline and 0.83 at follow-up.

#### 2.3.4 Epworth Sleepiness Scale (ESS)

The ESS is an eight-item self-report scale to assess the severity of daytime sleepiness (25). Each item is rated from 0 (never doze) to 3 (high chance of dozing). The total score ranges from 0 to 24, with a higher score indicating greater sleepiness. Cronbach’s alpha was 0.79 at baseline and 0.81 at follow-up.

#### 2.3.5 Fatigue Severity Scale (FSS)

The daytime fatigue severity was assessed using the FSS (26), which is a nine-item self-report scale. Each item is rated from 1 (disagreement) to 7 (strong agreement). FSS data are usually expressed as a mean score ranging from 1 to 7, with a higher score representing higher fatigue severity. Cronbach’s alpha was 0.85 at baseline and 0.82 at follow-up.

#### 2.3.6 Hypnotic medication use

The dose and frequency of hypnotic medication use at baseline and follow-up were collected via patient self-report. Hypnotics included benzodiazepines, non-benzodiazepines (Z-drugs), orexin receptor antagonists, histamine receptor antagonists, melatonin receptor agonists, and low-dose antidepressants (e.g., amitriptyline, doxepin, mianserin, mirtazapine, trazodone). The outcomes of hypnotic medication use were categorized as discontinued, decreased (dose or frequency), no change, and increased (dose or frequency).

### 2.4 Statistical analysis

The normality of continuous variables was assessed using the Shapiro−Wilk test. Normally distributed data are presented as the mean (standard deviation [SD]), and non-normally distributed data are presented as the median (interquartile range). Categorical variables are presented as frequency and percentage. The paired Wilcoxon signed-rank test and the chi-square test were used to identify differences from baseline to follow-up. The effect size (Cohen’s *d*) was calculated using the *Z* statistic obtained from Wilcoxon’s signed-rank test (27). The efficacy analysis was based on the intention-to-treat principle. Data from all patients were included in the statistical analysis. Missing data were imputed using the last observation carried forward method to make the results more conservative. We conducted a sensitivity analysis to determine the robustness of results for the scale outcomes. *P* values of ≤ 0.05 were considered statistically significant. All statistical analyses were performed using SPSS 27.0 for Windows (SPSS Inc., Chicago, IL, USA).

## 3 Results

**Figure 1:**
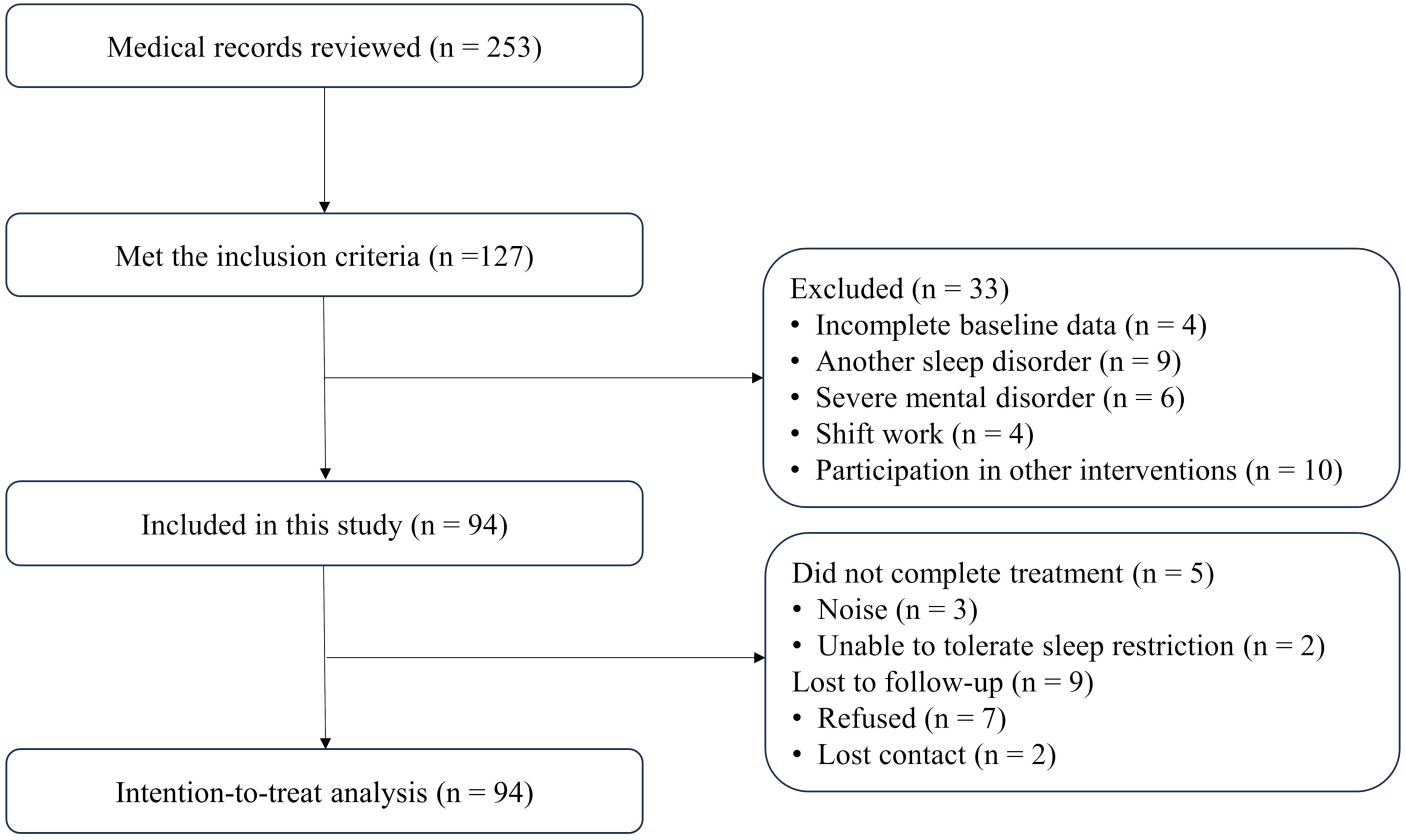
Flow diagram depicting patient inclusion and exclusion criteria in the study.

The medical records of 253 patients were reviewed and 127 met the inclusion criteria. Thirty-three patients were excluded based on exclusion criteria. A total of 94 patients were included in this study (**Figure 1**). Among them, 5 (5.3%) did not complete treatment and 9 (9.6%) were lost to follow-up.

### 3.1 Demographics

The mean age of the patients was 54.1 (SD 13.1) years (range 24–75 years). The majority of the patients were female (62.8%), married (91.5%), post-secondary education (51.1%), retired (51.1%), and taking hypnotic medication (78.7%) (**Table 2**).

**Table 2:** Demographic characteristics of the included patients (n = 94).

Variable	Value
Sex, n (%)	
Male	35 (37.2)
Female	59 (62.8)
Age (years), mean (SD)	54.1 (13.1)
Marital status, n (%)	
Single	8 (8.5)
Married	86 (91.5)
Education level, n (%)	
Primary	15 (16.0)
Secondary	31 (33.0)
Post-secondary	48 (51.1)
Working status, n (%)	
Employed	42 (44.7)
Unemployed	4 (4.3)
Retired	48 (51.1)
Medication taking, n (%)	
Hypnotics	58 (61.7)
Hypnotics + antidepressants and/or antipsychotics	16 (17.0)

SD, standard deviation.

### 3.2 Sleep and daytime functioning

**Table 3** displays the change in scale scores from baseline to the follow-up assessment. Significant improvements were noted in all domains, including insomnia severity (*Z* = −7.65, *P* < 0.001, Cohen’s *d =* 1.34, anxiety (*Z* = −6.23, *P* < 0.001, Cohen’s *d =* 1.02), depression (*Z* = −6.42, *P* < 0.001, Cohen’s *d =* 1.06), daytime sleepiness (*Z* = −2.40, *P* = 0.016, Cohen’s *d =* 0.35), and fatigue severity (*Z* = −5.54, *P* < 0.001, Cohen’s *d =* 0.88). The effect sizes were small for daytime sleepiness and large for all other domains from baseline to the follow-up assessment.

**Table 3:** Changes in self-report scale scores from baseline to follow-up (n = 94).

	Baseline Median (IQR)	Follow-up Median (IQR)	Median difference (95% CI)	*Z*	*P*	Cohen’s *d*
ISI	18.0 (15.0–21.3)	7.0 (2.0–14.0)	−10.0 (−11.5 to −8.0)	−7.65	< 0.001	1.34
HADS-A	6.0 (3.0–8.3)	2.0 (0.0–6.0)	−3.0 (−3.5 to −2.0)	−6.23	< 0.001	1.02
HADS-D	8.0 (4.0–12.0)	3.0 (0.0–7.3)	−3.5 (−4.5 to −2.5)	−6.42	< 0.001	1.06
ESS	5.0 (1.8–8.0)	3.0 (1.0–7.0)	−1.0 (−2.5 to 0.0)	−2.40	0.016	0.35
FSS	4.1 (2.3–5.3)	2.1 (1.1–4.0)	−1.2 (−1.6 to −0.7)	−5.54	< 0.001	0.88

CI, confidence interval; ESS, Epworth Sleepiness Scale; FSS, Fatigue Severity Scale; HADS-A, Hospital Anxiety and Depression Scale-Anxiety; HADS-D, Hospital Anxiety and Depression Scale-Depression; IQR, interquartile range; ISI, Insomnia Severity Index.

At the follow-up assessment, 58 patients (67.4%) demonstrated clinically meaningful changes in insomnia (decrease in ISI score of >8) and were considered as treatment responders. Fifty-one patients (59.3%) met the criteria for insomnia remission (ISI score < 8), and six (7.0%) experienced insomnia symptom exacerbation (increase in ISI score).

### 3.3 Efficacy of hypnotic medication discontinuation

At baseline, 74 patients (78.7%) were taking hypnotic medication, which decreased to 32 patients (34.0%) at follow-up (*χ^2^* = 33.62, *P* < 0.001). Specifically (**Table 4**), of the 74 patients taking hypnotic medication at baseline, 46 (62.2%) discontinued, 6 (8.1%) decreased, 2 (2.7%) increased, and 20 (27.0%) had no change at follow-up. Of the 20 patients who were not taking hypnotic medication at baseline, 4 (20.0%) were taking hypnotic medication at follow-up and 16 (80.0%) had no change.

**Table 4:** Changes in hypnotic medication use.

Baseline	Follow-up			
	Discontinued	Decreased	No change	Increased
Taking (n = 74)	46	6	20	2
Not taking (n= 20)	n/a	n/a	16	4

n/a, not applicable.

### 3.4 Sensitivity analysis

In the sensitivity analysis, we conducted intention-to-treat analysis with median imputation method (n = 94) and complete-case analysis (n = 85). The sensitivity analysis showed similar results, albeit of a higher magnitude of effect sizes in all of the outcomes (**Supplementary Tables S1**, **S2**).

The sensitivity analysis showed that the results were robust.

## 4 Discussion

In this retrospective study, we investigated the effectiveness of one-week inpatient CBT-I for patients with insomnia without severe mental disorders. The results showed that one-week inpatient CBT-I significantly improved insomnia and daytime symptoms and reduced hypnotic medication use. The effect was maintained at 3 months after treatment. These results are consistent with previous studies on CBT-I delivered in other formats or with other treatment durations (28–31). Although this study was uncontrolled, the effect sizes for most of the outcomes were large. Spontaneous fluctuations are unlikely to account for the improvement after treatment. In addition, the dropout rate was very low (5.3%), suggesting that inpatient CBT-I had good acceptance. Our findings provide preliminary support for short-term inpatient CBT-I, indicating that abbreviated inpatient CBT-I may be feasible and has therapeutic potential.

The unique contribution of this program is in controlling the cost of inpatient CBT-I. We shortened the hospitalization duration to 1 week and held group sessions (but not individual sessions) to teach patients the core knowledge of CBT-I. In our department, the mean inpatient bed-day based expenses of insomnia disorder was about 800 *RMB*. Thus, we estimate this program could save approximately 5,600 *RMB* in hospitalization costs compared to traditional 14-day programs. The CBT-I providers were therapists, not licensed clinicians. The patients were not required to receive regular outpatient treatment and provide their sleep diaries. Despite our abbreviated inpatient CBT-I program, the treatment outcomes remained satisfactory. Future studies should examine the effects of shorter hospitalization durations or sessions provided by nurses to further reduce costs. In addition, future studies should also investigate the direct costs (the cost of treatment to payers) and indirect costs (e.g., healthcare utilization, unbale to work) of inpatient CBT-I to determine the incremental cost-effectiveness ratio. The effects on patients with insomnia with comorbidities could also be studied to extend the use of inpatient CBT-I in stepped-care.

Demographically, the majority of the patients in our sample were retired (51.1%) and taking hypnotic medication (78.7%), with the rates being much higher than in previous studies of the general population (32–34). It is understandable that patients who are retired have more discretionary time and are more prone to hospitalization than patients who are employed. Patients who expect to discontinue hypnotic medication or who have difficulty discontinuing hypnotic medication may prefer inpatient CBT-I for additional medical support.

The reason we chose group therapy is that it is considered to be as effective as individual therapy, but it is more cost- and time-efficient (13, 35). In addition, group therapy allows individuals to connect with others going through the same issues, fostering a sense of community and understanding. Patients often prefer the opportunity to interact with peers who are also experiencing insomnia (36). The patients who preferred face-to-face services credited that the therapeutic effectiveness of CBT-I was mainly due to the interaction with the clinician, rather than the therapy *per se* (36, 37). Inpatient CBT-I can provide patients with more opportunities to interact with their peers and clinicians, which is the advantage of inpatient CBT-I.

Nevertheless, implementation of inpatient CBT-I in public hospitals is challenging. One of the challenges is environmental noise. We acknowledge that due to the limited medical resources in China, it is impossible to place patients in single-person rooms. Unsynchronized bedtimes, rolling over or snoring, and air conditioning noise can contribute to a poor sleep experience. Three patients (3.2%) dropped out of the treatment due to noise in the present study. The other challenge is the limited opportunity for activities. We were unable to provide adequate space and facilities for the patients to perform activities, which may have hindered the patients from accumulating sleep pressure.

This study has some limitations that should be considered. First, the design of this study was retrospective and uncontrolled, limiting the conclusions that could be drawn. Further randomized controlled trials are necessary. Second, patients were self-referred or referred to the CBT-I program by specialists to the, which could lead to selection bias. Third, we did not conduct a cost-effectiveness analysis to determine whether the cost of inpatient CBT-I outweighed the reduction in socioeconomic cost. Fourth, group session workshops may not adequately address the specific problems of individuals. A delivery format that combines group and individual sessions may be a useful addition.

In conclusion, our preliminary results imply that one-week inpatient CBT-I may be an effective intervention for the treatment of insomnia in patients without severe mental disorders by decreasing the severity of insomnia, enhancing daytime functioning, and facilitating the discontinuation of hypnotic medication. Inpatient CBT-I may be abbreviated and become an option in the management of stepped-care.

## Data Availability

The raw data supporting the conclusions of this article will be made available by the authors, without undue reservation.
