# Supplementary material for: One-week inpatient cognitive behavioral therapy for insomnia: a retrospective study
Source: Front Psychiatry. 2024 Aug 27;15:1440026. doi: 10.3389/fpsyt.2024.1440026 (PMC11384567; doi:10.3389/fpsyt.2024.1440026)
Supplement: Supplementary file 1 [file Table1.docx]

**Supplementary material**

**Table S1.** Intention-to-treat analysis with median imputation method for changes in self-report scale scores (n = 94)

|  | Baseline  Median (IQR) | Follow-up  Median (IQR) | Median difference  (95% CI) | *Z* | *P* | Cohen’s*d* |
| --- | --- | --- | --- | --- | --- | --- |
| ISI | 18.0 (15.0–21.3) | 7.0 (2.0–11.0) | −11.0 (−12.5 to −9.5) | −8.10 | < 0.001 | 1.46 |
| HADS-A | 6.0 (3.0–8.3) | 1.0 (0.0–5.0) | −3.5 (−4.0 to −2.5) | −6.71 | < 0.001 | 1.12 |
| HADS-D | 8.0 (4.0–12.0) | 3.0 (0.0–6.0) | −4.0 (−5.0 to −3.0) | −6.83 | < 0.001 | 1.15 |
| ESS | 5.0 (1.8–8.0) | 3.0 (1.0–6.0) | −1.5 (−2.5 to −0.5) | −2.74 | 0.006 | 0.41 |
| FSS | 4.1 (2.3–5.3) | 2.1 (1.1–3.4) | −1.4 (−1.8 to −0.9) | −5.94 | < 0.001 | 0.96 |

CI, confidence interval; ESS, Epworth Sleepiness Scale; FSS, Fatigue Severity Scale; HADS-A, Hospital Anxiety and Depression Scale-Anxiety; HADS-D, Hospital Anxiety and Depression Scale-Depression; IQR, interquartile range; ISI, Insomnia Severity Index.

**Table S2.** Complete-case analysis for changes in self-report scale scores (n = 85)

|  | Baseline  Median (IQR) | Follow-up  Median (IQR) | Median difference  (95% CI) | *Z* | *P* | Cohen’s*d* |
| --- | --- | --- | --- | --- | --- | --- |
| ISI | 18.0 (15.0–21.5) | 7.0 (2.0–13.0) | −11.0 (−12.5 to −9.0) | −7.65 | < 0.001 | 1.45 |
| HADS-A | 6.0 (3.0–8.0) | 1.0 (0.0–5.0) | −3.0 (−4.0 to −2.5) | −6.23 | < 0.001 | 1.09 |
| HADS-D | 8.0 (4.0–12.5) | 3.0 (0.0–7.0) | −3.5 (−5.0 to −2.5) | −6.42 | < 0.001 | 1.13 |
| ESS | 5.0 (2.0–8.0) | 3.0 (1.0–6.0) | −1.5 (−2.5 to −0.5) | −2.40 | 0.016 | 0.38 |
| FSS | 4.0 (2.4–5.1) | 2.1 (1.1–3.6) | −1.3 (−1.7 to −0.9) | −5.54 | < 0.001 | 0.94 |

CI, confidence interval; ESS, Epworth Sleepiness Scale; FSS, Fatigue Severity Scale; HADS-A, Hospital Anxiety and Depression Scale-Anxiety; HADS-D, Hospital Anxiety and Depression Scale-Depression; IQR, interquartile range; ISI, Insomnia Severity Index.
